# *Gynoxys reinaldii* Cuatrec. and *Gynoxys pulchella* (Kunth) Cass.: Chemical and Enantioselective Analyses of Two Unprecedented Essential Oils from Ecuador

**DOI:** 10.3390/plants13243543

**Published:** 2024-12-19

**Authors:** Yessenia E. Maldonado, María del Carmen Rodríguez, Karyna Calvopiña, Omar Malagón, Nixon Cumbicus, Gianluca Gilardoni

**Affiliations:** 1 Programa de Doctorado en Química, Universidad Técnica Particular de Loja (UTPL), Calle Paris s/n y Praga, Loja 110107, Ecuador; yemaldonado2@utpl.edu.ec; 2 Carrera de Bioquímica y Farmacia, Universidad Técnica Particular de Loja (UTPL), Calle Paris s/n y Praga, Loja 110107, Ecuador; mdrodriguez15@utpl.edu.ec; 3 Departamento de Química, Universidad Técnica Particular de Loja (UTPL), Calle Paris s/n y Praga, Loja 110107, Ecuador; kcalvopinac@uteq.edu.ec (K.C.); omalagon@utpl.edu.ec (O.M.); 4 Facultad de Ciencias de la Ingeniería, Universidad Técnica Estatal de Quevedo (UTEQ), Campus Central, Av. Quito km 11/2 vía a Santo Domingo de los Tsáchilas, Quevedo 120301, Ecuador; 5 Departamento de Ciencias Biológicas y Agropecuarias, Universidad Técnica Particular de Loja (UTPL), Calle Paris s/n y Praga, Loja 110107, Ecuador; nlcumbicus@utpl.edu.ec

**Keywords:** asteraceae, mass spectrometry, enantiomeric composition, β-cyclodextrin, sesquiterpene

## Abstract

This study presents the first chemical and enantioselective analyses of essential oils (EOs) derived from the leaves of two endemic species, *Gynoxys reinaldii* Cuatrec. and *Gynoxys pulchella* (Kunth) Cass., from Loja, Ecuador. The distillation yields, by weight of dry plant material, were 0.04 ± 0.007% for *G. reinaldii* and 0.03 ± 0.002% for *G. pulchella*. For both plants, the chemical analyses were conducted by GC-MS (qualitative) and GC-FID (quantitative), on two stationary phases of different polarity (5% phenyl-methylpolysiloxane and polyethylene glycol). The major components of *G. reinaldii* EO included germacrene D (22.3–22.1%), α-pinene (14.2–14.1%), and (*E*)-β-caryophyllene (13.6–14.5%). Similarly, *G. pulchella* EO was characterized by germacrene D (9.5–12.9%), caryophyllene oxide (7.2–6.7%), and *n*-tricosane (4.9% in both columns). The enantioselective analyses were carried out with two columns, based on 2,3-diacetyl-6-*tert*-butyldimethylsilyl-β-cyclodextrin and 2,3-diethyl-6-*tert*-butyldimethylsilyl-β-cyclodextrin, detecting nine chiral terpenes and terpenoids. In *G. reinaldii* EO, (1*S*,5*S*)-(−)-α-pinene, (1*S*,5*S*)-(−)-β-pinene, (1*S*,5*S*)-(−)-sabinene, (*R*)-(−)-α-phellandrene, and (*R*)-(−)-β-phellandrene were enantiomerically pure, whereas *cis*-linalool oxide, linalool, terpinene-4-ol, and germacrene D were non-racemic mixtures of enantiomers. In *G. pulchella*, only (*R*)-(−)-α-phellandrene was enantiomerically pure. The detection of enantiomerically pure compounds may provide insights into the biosynthetic pathways and potential bioactivities of these EOs.

## 1. Introduction

Since ancient times, in all cultures worldwide, plants have been the main source of organic chemicals. Drugs, venoms, perfumes, and fats have been obtained from vegetal materials, usually as complex mixtures. Since the discovery of morphine, many pure natural products have been identified in botanical species, characterized by biological activities or physiological properties [1]. However, after two centuries of research, most of the biodiversity in Europe and North America has been investigated, obliging chemists to focus on tropical flora, especially in “megadiverse” countries like Ecuador [2].

Thanks to the novelty of the Ecuadorian flora, our group has been investigating the very wide but poorly studied chemical diversity of this country for more than 20 years [3,4]. Initially interested in discovering new non-volatile compounds, we recently focused also on the description of unprecedented essential oils (EOs), with an emphasis on their chemical and enantiomeric compositions, biological activity, and olfactory profile [5,6,7,8,9,10].

The main objective of the present study is to enhance the knowledge about phytochemistry and chemotaxonomy of genus *Gynoxys* Cass., which belongs to the family Asteraceae, in the province of Loja (Ecuador). This genus is native to Argentina, Bolivia, Colombia, Ecuador, Peru, and Venezuela, but the country with the most described specimens is Ecuador [11,12,13]. So far, seven Ecuadorian species have been described as a part of this unfunded project [14,15,16,17,18,19].

On the one hand, according to the literature, *G. reinaldii* is an endemic shrub, growing between 2000 and 3000 m above the sea level. It has been recorded in the provinces of Azuay and Loja [20]. On the other hand, *G. pulchella* is an endemic tree, growing at an altitude of 3500–4000 m, and recorded in the provinces of Bolívar, Tungurahua, and Loja. This species is also known as *Senecio pulchellus* Kunth [12,20]. These taxa have no reported medicinal use, and this study provides the first description of their essential oil composition and enantiomeric profile.

## 2. Results

### 2.1. Chemical Analyses of the EOs

**Figure 1:**
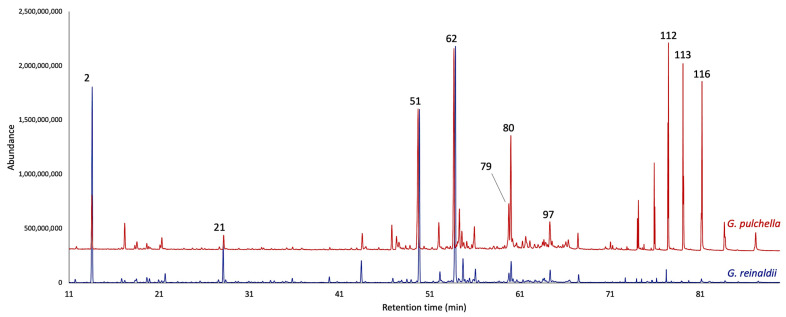
Compared GC-MS profiles of *G. reinaldii* (blue) and *G. pulchella* (red) EOs from on a 5% phenyl-methylpolysiloxane stationary phase. The numbers refer to peak numbers in Table 1.

**Figure 2:**
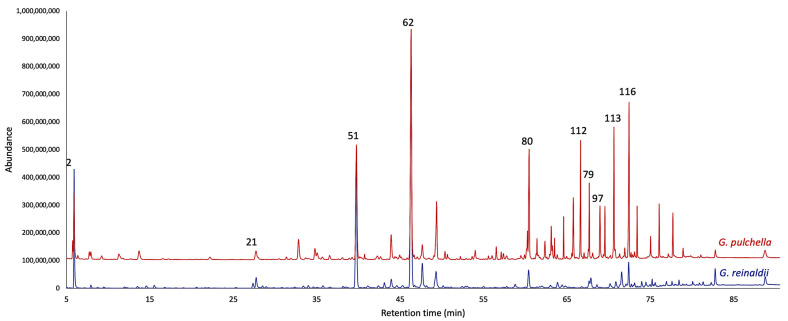
Compared GC-MS profiles of *G. reinaldii* (blue) and *G. pulchella* (red) EOs on a polyethylene glycol stationary phase. The numbers refer to peak numbers in Table 1.

The distillation yields, analytically calculated over four repetitions by weight of dry plant material, were 0.04 ± 0.007% for *G. reinaldii* and 0.03 ± 0.002% for *G. pulchella*. The EOs, analyzed on two stationary phases of different polarity, permitted to detect, and quantify a total of 123 compounds, of which only five resulted to be unidentified. In *G. reinaldii* EO, the total amount of quantified compounds corresponded to 86.3% and 82.8% of the total oil mass on a non-polar and polar column, respectively, whereas in *G. pulchella* EO, the quantified components altogether corresponded to 90.2% and 91.2%. As is usual for the genus *Gynoxys*, both essential oils were dominated by the sesquiterpene fraction, including hydrocarbons and oxygenated sesquiterpenoids. About *G. reinaldii*, the terpene fraction accounted for 54.7–53.4%, whereas *G. pulchella* EO showed 47.9–50.2%. On the one hand, for *G. reinaldii*, the second most abundant fraction was constituted by monoterpenes and oxygenated monoterpenoids, whose abundance was 18.7–17.2% on the two columns, respectively. On the other hand, the second main fraction of *G. pulchella* was composed of non-terpene compounds, principally heavy aliphatic hydrocarbons, whose amount was 34.2–34.3%. The main constituents of *G. reinaldii* EO (≥3.0 on at least one column) were germacrene D (22.3–22.1%, peak 62), α-pinene (14.2–14.1%, peak 2), (*E*)-β-caryophyllene (13.6–14.5%, peak 51), *n*-nonanal (3.0–2.3%, peak 21), and caryophyllene oxide (3.0–3.1%, peak 80). About *G. pulchella*, the major components were germacrene D (9.5–12.9%, peak 62), caryophyllene oxide (7.2–6.7%, peak 80), (*E*)-β-caryophyllene (7.0–7.8%, peak 51), *n*-tricosane (4.9% on both columns, peak 116), 1-docosene (4.0–4.3%, peak 113), α-pinene (3.7–3.0%, peak 2), spathulenol (3.6–3.2%, peak 79), *n*-heneicosane (3.6–3.5%, peak 112), and α-cadinol (2.1–3.0%, peak 97). The detailed analytical results are shown in Table 1, whereas the GC profiles are reported in Figure 1 and Figure 2.

### 2.2. Enantioselective Analyses

**Figure 3:**
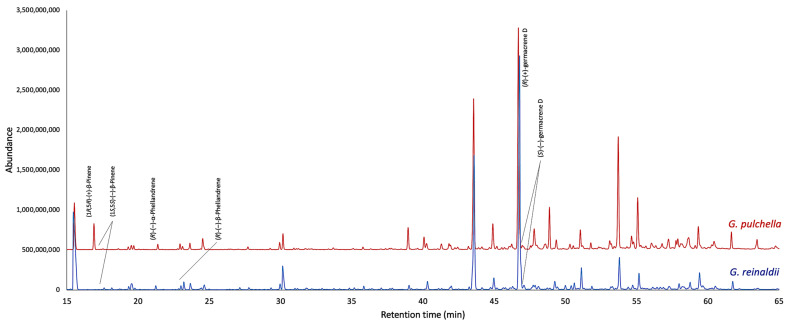
Compared GC-MS profiles of *G. reinaldii* (blue) and *G. pulchella* (red) EOs on a 2,3-diethyl-6-*tert*-butyldimethylsilyl-β-cyclodextrin stationary phase.

**Figure 4:**
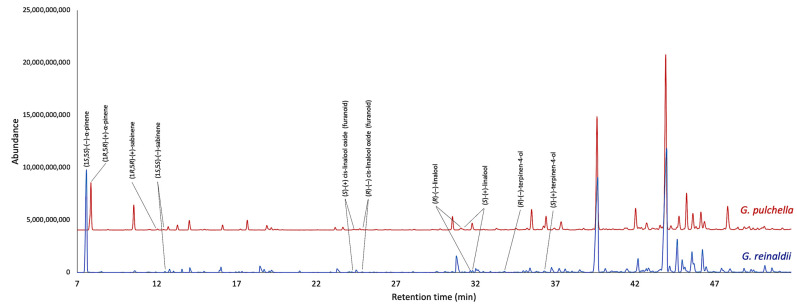
Compared GC-MS profiles of *G. reinaldii* (blue) and *G. pulchella* (red) EOs on a 2,3-diacethyl-6-*tert*-butyldimethylsilyl-β-cyclodextrin stationary phase.

The enantioselective analyses, depending on the EOs chemical composition and the availability of enantiomerically pure standards, were carried out on nine chiral terpenes and terpenoids, whose results are detailed in Table 2, whereas the GC profiles are represented in Figure 3 and Figure 4. In *G. reinaldii* EO, (1*S*,5*S*)-(−)-α-pinene, (1*S*,5*S*)-(−)-β-pinene, (1*S*,5*S*)-(−)-sabinene, (*R*)-(−)-α-phellandrene, and (*R*)-(−)-β-phellandrene were enantiomerically pure, whereas *cis*-linalool oxide, linalool, terpinene-4-ol, and germacrene D were scalemic mixtures, with linalool almost racemic. About *G. pulchella* EO, only (*R*)-(−)-α-phellandrene was enantiomerically pure. In both oils, as is usual in many *Gynoxys* species, germacrene D showed a very high enantiomeric excess (>96%).

## 3. Discussion

According to the literature, the genus *Gynoxys* comprises approximately 130 species, of which 34 are recorded in Ecuador and at least 23 are endemic [13,20]. In the present project, 12 species were selected in the province of Loja to be submitted to EO analysis: *Gynoxys miniphylla* Cuatrec., *Gynoxys rugulosa* Muschl., *Gynoxys buxifolia* (Kunth) Cass., *Gynoxys laurifolia* (Kunth) Cass., *Gynoxys cuicochensis* Cuatrec., *Gynoxys sancti-antonii* Cuatrec., *Gynoxys szyszylowiczii* Hieron., *Gynoxys calyculisolvens* Hieron., *Gynoxys hallii* Hieron., *Gynoxys azuayensis* Cuatrec., *Gynoxys pulchella* (Kunth) Cass., *Gynoxys reinaldii* Cuatrec. So far, the EOs of *G. miniphylla*, *G. rugulosa*, *G. buxifolia*, *G. laurifolia*, *G. cuicochensis*, *G. sancti-antonii*, and *G. szyszylowiczii* have been described and published, whereas *G. calyculisolvens*, *G. hallii*, and *G. azuayensis* are being investigated. Finally, *G. pulchella* and *G. reinaldii* are the subjects of the present study [14,15,16,17,18,19].

**Figure 5:**
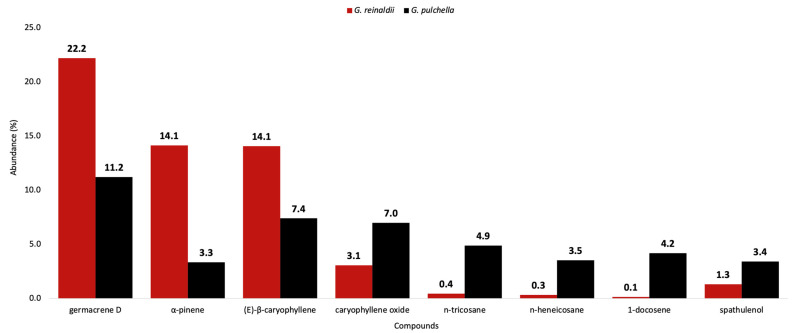
Compared abundance of major compounds (≥3.0 in at least one oil) in the EOs of *G. reinaldii* (red) and *G. pulchella* (black). Abundances correspond to the mean values of the quantitative results with both columns.

The EOs distilled from the leaves of *G. reinaldii* and *G. pulchella* presented quite similar chemical profiles, typical of the volatile fractions of this genus. In fact, as is usual for the *Gynoxys* spp., three main fractions can be recognized: a poor monoterpene fraction with a strong presence of α-pinene, a dominant sesquiterpene fraction, and a heavy aliphatic fraction [14,15,16,17,18,19]. In Figure 5, the abundances of the main components in these EOs are compared, expressed as mean percent values on the two columns. Germacrene D is the most abundant compound in both oils, whereas (*E*)-β-caryophyllene is the second main component in *G. pulchella* and the third one in *G. reinaldii*. On the other hand, α-pinene is the second major constituent in *G. reinaldii*. Compositions where these terpenes were present as main components have already been observed in other EOs from this genus, e.g., in the case of *G. rugulosa*, *G. laurifolia*, *G. szyszylowiczii*, and *G. cuicochensis* [15,17,18,19]. In *G. miniphylla*, the main EO component is α-phellandrene, whereas in *G. sancti-antonii*, it is γ-curcumene. However, in all cases, germacrene D, α-pinene, and (*E*)-β-caryophyllene are present in relatively high amounts [14,15,16,17,18,19]. Regarding the heavy aliphatic fraction, this is very important in *G. pulchella*, but almost negligible in *G. reinaldii*. However, other *Gynoxys* spp. were characterized by an abundant fraction, as it is the case for *G. rugulosa* and *G. szyszylowiczii* [15,19]. Anyway, as a trace or in low amount, most of the analyzed *Gynoxys* EOs presented heavy alkanes and alkenes, confirming that the corresponding biosynthetic pathway is common within this genus.

**Figure 6:**
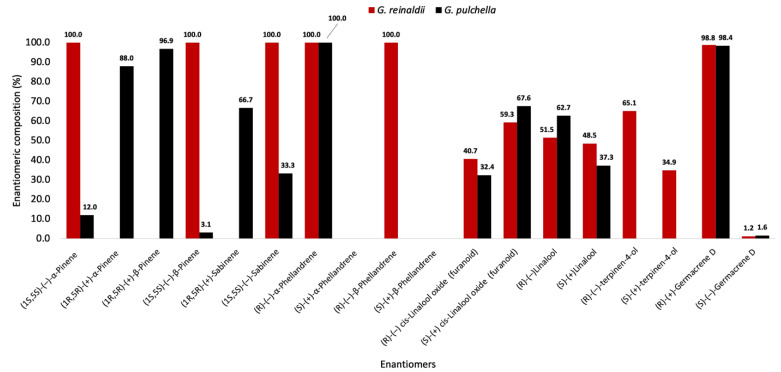
Compared enantiomeric composition of some chiral compounds in the EOs of *G. reinaldii* (red) and *G. pulchella* (black).

For what concerns the enantiomeric composition of these EOs, a graphical comparison between the two is represented in Figure 6. Unlike the chemical analyses, the enantiomeric profiles were not so similar, with some radical differences among the hydrocarbon monoterpenes. In fact, it can be observed that G. *reinaldii* produced enantiomerically pure (1*S*,5*S*)-(−)-α-pinene and (1*S*,5*S*)-(−)-β-pinene, whereas *G. pulchella* presented high enantiomeric excesses of both dextrorotatory forms. A similar but not identical trend could be observed for sabinene. On the other hand, both species produced the same enantiomer of α-phellandrene, whereas β-phellandrene is absent in *G. pulchella* and its laevorotatory form is enantiomerically pure in *G. reinaldii*. These results about pinenes and phellandrenes are somehow biosynthetically consistent. In fact, the stereogenic centres in pinenes are formed when the pinyl cation, the direct precursor of both α-pinene and β-pinene, is produced [80]. Similarly, both α-phellandrene and β-phellandrene derive from the same phellandryl cation, sharing the configuration of the only asymmetric carbon [80]. Although a different trend has sometimes been observed, the expected situation is as described here: the same absolute configuration for α- and β-pinene on one side, and α- and β-phellandrene on the other side, within the same species. About linalool and linalool oxide, the two EOs were instead similar. Not only were these enantiomeric mixtures relatively close to racemic, but, also, in both plants, the enantiomeric excess of linalool oxide was in favor of the dextrorotatory form, whereas for linalool, the laevorotatory isomer was dominant. This phenomenon suggested that linalool oxide could be obtained from linalool through an enantiospecific oxidation. Finally, (*R*)-(+)-germacrene D presented a very high enantiomeric excess in both plants. This condition, where germacrene D is almost enantiomerically pure, is common in the genus *Gynoxys*, although sometimes the dominant isomer is dextrorotatory, and at other times, laevorotatory.

**Figure 7:**
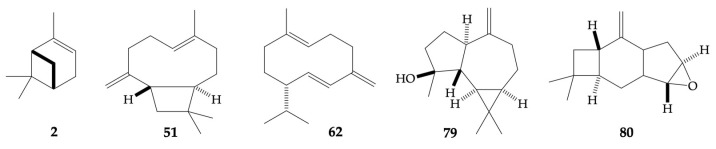
Major terpene constituents of *G. reinaldii* and *G. pulchella* EOs (≥3.0 in at least one oil, as a mean value on both columns). The numbers refer to Table 1: α-pinene (**2**), (*E*)-β-caryophyllene (**51**), germacrene D (**62**), spathulenol (**79**), and caryophyllene oxide (**80**).

About a possible biological activity of these EOs, the analytical scale distillation did not permit to carry out any assay with this respect. In fact, in the analytical approach, the EO is distilled over an exact volume of cyclohexane, containing an internal standard. The condensed vapors are extracted through the solvent, avoiding the obtention of a pure volatile fraction. The obtained cyclohexane solution can be directly injected into GC, however this approach usually prevents from any application that requires a pure EO, such as conducting a biological activity test. Nevertheless, some literature exists on the properties of the five major terpene constituents (see Figure 7), whose biological activities can be reflected in *G. reinaldii* and *G. pulchella* EOs. For what concerns germacrene D (**62**), the main property reported is an ecologically interesting attractive effect for the moths of the genus *Heliothis* and *Helicoverpa* [81]. However, according to further studies, it seems to be the laevorotatory enantiomer the one responsible for this capacity, whereas *G. reinaldii* and *G. pulchella* almost exclusively produced (*R*)-(+)-germacrene D [82,83]. If the role of (*R*)-(+)-germacrene D versus *Heliothis* and *Helicoverpa* spp. can be excluded, the ecological properties of other abundant terpenes and terpenoids must be mentioned. This is for instance the case of α-pinene (**2**), (*E*)-β-caryophyllene (**51**), and caryophyllene oxide (**80**), whose antifungal, allelopathic, insect-repellent, and insect-attractive effects have been known for more than 40 years [84]. In particular, on the one hand, caryophyllene oxide (**80**) demonstrated a good fungistatic activity against *Pestalotia subcuticularis*, a leaf-spotting fungi, pathogen to genus *Hymenaea*. On the other hand, (*E*)-β-caryophyllene (**51**), whose fungistatic activity was very modest, showed important insecticidal capacity against herbivorous lepidoptera, also threatening the genus *Hymenaea*. If we consider that *Hymenaea* spp. produce both compounds, whose relative abundance depends on the ecological necessity of the plant, a similar role could be hypothesized for these metabolites in *G. reinaldii* and *G. pulchella* EOs.

After germacrene D, the second main component of *G. reinaldii* EO was α-pinene (**2**), probably one of the pharmacologically most investigated terpenes. This metabolite is known for possessing antibacterial, antifungal, anti-leishmanial, anti-inflammatory, antioxidant, neuroprotective, antitumor, insecticidal, and nematocidal activities, with the anti-inflammatory capacity likely being the most important. As expected, its enantiomers present different biological properties, with (1*S*,5*S*)-(−)-α-pinene (*G. reinaldii*) being anti-viral, whereas (1*R*,5*R*)-(+)-α-pinene (*G. pulchella*) is neuroprotective [85,86]. After that, (*E*)-β-caryophyllene (**51**) was the third main constituent in both oils, almost reaching 15% in *G. reinaldii* (similar to α-pinene) and 7% in *G. pulchella* (similar to caryophyllene oxide). This very common sesquiterpene hydrocarbon has been widely studied, and its anti-inflammatory, neuroprotective, analgesic, antioxidant, sedative, anxiolytic, and antitumor activities have been described. However, also in this case, the anti-inflammatory and anticancer capacities probably are the most important [87,88]. Caryophyllene oxide (**80**) was as abundant as (*E*)-β-caryophyllene in *G. pulchella* EO. This oxygenated sesquiterpenoid is an epoxide, directly deriving from the oxidation of (*E*)-β-caryophyllene, with which it shares anticancer activity. This property seems to be more evident in caryophyllene oxide than in (*E*)-β-caryophyllene, and it has been explained by the electrophilic character of the epoxide group [89]. Furthermore, both (*E*)-β-caryophyllene and caryophyllene oxide showed an interesting analgesic activity as a consequence of their affinity for the CB2 cannabinoid receptors [89].

Finally, spathulenol (**79**) was a major oxygenated terpenoid in the EO of *G. pulchella*. Although this compound has not been extensively studied in a pure form about its pharmacology, there is a significant amount of literature on the biological activities of EOs where spathulenol is the most abundant constituent. This is, for example, the case of *Psidium guineense* Sw., where spathulenol accounted for more than 80% of the total oil composition. On that occasion, both EO and purified spathulenol demonstrated important anti-inflammatory activity, as well as moderate antiproliferative and antimycobacterial capacities against an ovarian cancer cell line and *Mycobacterium tuberculosis,* respectively [90].

As is usual within natural products, the presence of both enantiomerically pure or scalemic chiral compounds is the result of enantioselective and enantiospecific biosynthetic pathways. The need for a specific chirality in secondary metabolites depends on the different biological properties associated with different enantiomers. In fact, it is well known that, although two optical isomers are characterized by the same physicochemical properties (except for optical activity), they present different biological capacities due to the chiral medium constituted by living organisms (chiral receptors, chiral active sites, etc.). Therefore, when two enantiomers are produced by the same organism in nature, they usually come from different enantioselective biosynthetic pathways in order to play different biochemical roles.

## 4. Materials and Methods

### 4.1. Plant Material

The leaves of *G. reinaldii* were collected on 28 October 2022, from different shrubs, located in a range of 100 m around a central point of coordinates 03°59′42″ S and 79°16′10″ W, at an altitude of about 2600 m above the sea level. About *G. pulchella*, the leaves were harvested on June 6, 2023, in a range of about 100 m from a point of coordinates 03°41′36″ S and 79°17′49″ W, at about 3140 m above the sea level. The taxonomic identification was carried out by one of the authors (N.C.), based on the specimens conserved at the Missouri Botanical Garden with codes 3,595,614 (*G. reinaldii*) and 5,813,849 (*G. pulchella*). Two botanical vouchers were deposited at the herbarium of the Universidad Técnica Particular de Loja, with codes 14,665 and 14,674 for *G. reinaldii* and *G. pulchella,* respectively. Both collection places were located in the province of Loja, Ecuador. After collection, the plant materials were dried at 35 °C for 48 h and stored in dark bags until use. This investigation was conducted in compliance of Ecuadorian law, under the permission of the Ministry of Environment, Water, and Ecological Transition of Ecuador (MAATE), with registry code MAATE-DBI-CM-2022-0248. For both *G. reinaldii* and *G. pulchella*, the balsamic period was unknown, and the date of collection was not chosen as a function of the chemical profile or the distillation yield. The determination of the balsamic period would need, for each species, a year-long study that would go beyond the objectives of the present project. For the same reasons, no control has been applied to leaf age so far.

### 4.2. EOs Distillation and Samples Preparation

Both plants were analytically steam-distilled in four repetitions as previously described in the literature, i.e., using a modified Dean–Stark apparatus. Each repetition was obtained by distilling the plants over 2 mL of cyclohexane containing *n*-nonane as an internal standard [15]. Both solvent and internal standard were purchased from Merk (Sigma–Aldrich, St. Louis, MO, USA). A total of four samples for each species were obtained, which could be directly injected into the GC. In the distillation of *G. reinaldii*, each repetition was carried out from 80.0 g of dry plant material, whereas 84.4, 81.6, 51.6, and 52.0 g of dry leaves were used for *G. pulchella*. All the cyclohexane solutions were stored at −15 °C until use. The distillation apparatus was assembled with commercial glassware, and it was the same equipment used during the whole project for all the *Gynoxys* spp. Therefore, although it was not the result of a specific design (except for the modified Dean–Stark), it ensured the same reproducible physical parameters and conditions for all the investigated species. Similarly, the distillation yield was not experimentally optimized, but the distillation time was based on the authors’ experience and was maintained the same for all the species in this project.

### 4.3. Qualitative Analyses (GC-MS) of the EOs

The qualitative analyses were conducted on a Thermo Fisher gas chromatograph (GC) model Trace 1310, coupled with a ISQ 7000 mass spectrometer (MS) from the same provider (Thermo Fisher Scientific, Walthan, MA, USA). The oven was configured with two capillary columns based on the stationary phases of different polarity (30 m long, 0.25 mm internal diameter, and 0.25 μm film thickness). All the analyses were carried out on both phases, 5% phenyl-methylpolysiloxane (TR-5MS, non-polar) and polyethylene glycol (TR-Wax, polar), respectively, provided by Thermo Fisher Scientific (Walthan, MA, USA). The carrier gas was helium, purchased from Indura (Guayaquil, Ecuador), and set at the constant flow of 1 mL/min. All the elutions were conducted according to the following thermal gradient: 50 °C for 10 min, followed by a first gradient of 3 °C/min until 100 °C, a second gradient of 5 °C/min until 200 °C, and a third gradient of 10°C/min until 230 °C, which was maintained for 20 min. The injector was set at 230 °C and operated in split mode (40:1). The MS electron impact (EI) ion source was set at 70 eV and 250 °C, with the mass analyzer operating in SCAN mode (mass range 40–400 *m*/*z*), programmed at 250 °C. The transfer line temperature was 230 °C and the injection volume was 1 μL for all samples. Each component of the EOs was identified by comparison of its mass spectrum and linear retention index (LRI) with data from the literature. The LRIs were calculated according to Van den Dool and Kratz, referring to a mixture of homologous *n*-alkanes in the range C_9_–C_28_, purchased from Merk (Sigma–Aldrich, St. Louis, MO, USA) [91]. The use of two stationary phases of different polarity ensured that practically all the detected compounds could be separated with at least on column. Anyway, even when two constituents coeluted on a column, the identification was often successful, thanks to ion mass extraction.

### 4.4. Quantitative Analyses (GC-FID) of the EOs

The quantitative analyses were conducted with the same GC instrument, columns, thermal program, and conditions as the qualitative ones, but with a flame ionization detector (FID) instead of MS. The split value in all GC-FID analyses was 10:1. With each column, the EOs components were quantified using isopropyl caproate in a six-point calibration curve, where the six dilutions were prepared as previously described in the literature [92]. Before applying the integration areas to the calibration curves, a relative response factor (RRF) was calculated for each compound based on their combustion enthalpy [93,94]. The calibration standard (isopropyl caproate) was synthetized in the authors’ laboratories and purified to 98.8% (GC-FID). The internal standard was *n*-nonane, purchased from Merk (Sigma–Aldrich, St. Louis, MO, USA). Both calibration curves showed a correlation coefficient >0.998. Because of its dependence from the combustion enthalpy, the RRF value is practically the same for all isomers. Therefore, with FID, an isomer can be used instead of an original compound as a quantification standard. For this reason, we decided to use isopropyl caproate instead of methyl octanoate, which was originally employed by Alain Chaintreau in his method [93].

### 4.5. Enantioselective Analyses

The enantiomeric compositions of the EOs were determined using two different enantioselective columns, purchased from Mega, Milan, Italy. The column dimensions were 25 m in length, 0.25 mm in internal diameter, and 0.25 μm in phase thickness, whereas the chiral selectors were 2,3-diacetyl-6-*tert-*butyldimethylsilyl-β-cyclodextrin and 2,3-diethyl-6-*tert*-butyldimethylsilyl-β-cyclodextrin. The enantioselective analyses were carried out in the same GC-MS instrument and with the same MS settings previously described in Section 4.3, but with the following thermal program: 50 °C for 1 min, followed by a thermal gradient of 2 °C/min until 220 °C, which was maintained for 10 min. The injector and transfer line temperatures were set to 220 °C, whereas the carrier gas flow was set to a constant pressure of 70 kPa. The split value was 40:1, and the injection volume was 1 μL. The enantiomers were identified for their mass spectrum and with the aim of enantiomerically pure standards, injected in the same conditions. A mixture of *n*-alkanes (C_9_–C_28_), provided by Merk (Sigma–Aldrich, St. Louis, MO, USA), was also injected in the same conditions to calculate the retention indices. The chiral selectors were chosen according to the enantiomers that had to be separated and for which enantiomerically pure standards were available.

## 5. Conclusions

The leaves of *Gynoxys reinaldii* Cuatrec. and *Gynoxys pulchella* (Kunth) Cass. produced an EO with a distillation yield of 0.04 ± 0.007% and 0.03 ± 0.002%, respectively. On the one hand, the chemical compositions were relatively similar regarding the terpene fractions, but they were substantially different in the heavy aliphatic fractions. On the other hand, the enantiomeric compositions differed, and, as is usual for the EO of this genus, a common trend was not evident. The only exceptions were linalool and linalool oxide, whose respective enantiomeric compositions were consistent with the hypothesis of an enantiospecific oxidation. According to the chemical compositions, these EOs could be characterized by many biological properties, with the anti-inflammatory activity likely being the most important. Once the present project is complete, all the volatile fractions will be compared using proper statistical analysis.

## Figures and Tables

**Table 1:** Qualitative and quantitative compositions of *G. reinaldii* and *G. pulchella* EOs.

N°	Compounds	5% Phenyl-Methylpolysiloxane						Polyethylene Glycol						
		LRI		*G. reinaldii*		*G. pulchella*		LRI		*G. reinaldii*		*G. pulchella*		Lit.
		Calc.	Ref. [21]	%	σ	%	σ	Calc.	Ref.	%	σ	%	σ	
1	heptanal	911	901	0.3	0.10	-	-	1180	1180	0.4	0.08	-	-	[22]
2	α-pinene	934	932	14.2	3.81	3.7	0.77	1015	1015	14.1	3.57	3.0	0.55	[23]
3	α-fenchene	950	945	0.1	0.02	-	-	1053	1048	trace	-	-	-	[24]
4	thuja-2,4(10)-diene	955	953	0.1	0.00	-	-	1116	1116	0.3	0.08	-	-	[25]
5	sabinene	974	969	0.3	0.08	0.1	0.02	1113	1114	trace	-	0.1	0.03	[26]
6	β-pinene	978	974	0.2	0.05	2.0	0.46	1101	1102	0.2	0.05	1.7	0.34	[27]
7	myrcene	992	988	0.1	0.03	0.2	0.05	1159	1159	0.1	0.03	0.2	0.04	[28]
8	2-pentyl furan	994	984	0.6	0.15	0.6	0.10	1228	1229	0.5	0.08	0.2	0.10	[29]
9	*n*-decane	1000	1000	0.2	0.10	-	-	1000	1000	trace	-	-	-	-
10	α-phellandrene	1008	1002	0.3	0.06	0.6	0.08	1154	1153	0.3	0.09	0.2	0.04	[23]
11	*n*-octanal	1012	1017	0.4	0.13	0.4	0.10	1295	1295	0.2	0.04	trace	-	[30]
12	(2*E*,4*E*)-heptadienal	1024	1005	0.5	0.08	0.1	0.02	1482	1481	0.9	0.11	0.1	0.01	[31]
13	*p*-cymene	1029	1022	0.3	0.16	0.7	0.14	1260	1254	0.1	0.02	0.5	0.11	[32]
14	limonene	1031	1024	0.1	0.05	-	-	1188	1189	0.1	0.01	-	-	[33]
15	β-phellandrene	1033	1025	0.8	0.20	-	-	1197	1197	0.6	0.17	-	-	[34]
16	(*E*)-β-ocimene	1050	1044	0.1	0.03	-	-	1248	1246	trace	-	-	-	[33]
17	*cis*-linalool oxide (furanoid)	1075	1067	0.2	0.02	0.1	0.01	1461	1465	trace	-	trace	-	[26]
18	*n*-octanol	1081	1063	0.2	0.03	-	-	1555	1555	0.2	0.02	-	-	[35]
19	*cis*-vertocitral C	1088	1076	-	-	0.1	0.01	1206	-	-	-	trace	-	-
20	linalool	1106	1095	0.3	0.02	0.2	0.02	1548	1547	0.3	0.02	0.4	0.26	[36]
21	*n*-nonanal	1113	1100	3.0	0.59	1.0	0.09	1386	1387	2.3	0.38	0.7	0.12	[37]
22	*cis*-β-terpineol	1131	1140	0.1	0.02	-	-	1590	1639	0.1	0.01	-	-	[38]
23	α-campholenal	1135	1122	0.1	0.04	0.1	0.03	1475	1472	0.2	0.05	trace	-	[39]
24	citronellal	1150	1148	0.2	0.06	-	-	-	-	-	-	-	-	-
25	verbenol	1154	1140	0.2	0.04	-	-	-	-	-	-	-	-	-
26	isomer of compound 29	1159	1166	0.1	0.05	-	-	1653	-	0.3	0.03	-	-	-
27	ethyl benzoate	1167	1169	0.1	0.03	-	-	-	-	-	-	-	-	-
28	(2*E*)-nonen-1-al	1171	1157	0.2	0.03	-	-	1523	1524	0.2	0.02	-	-	[40]
29	*p*-mentha-1,5-dien-8-ol	1182	1185	0.3	0.05	-	-	1718	1719	0.3	0.06	-	-	[41]
30	terpinen-4-ol	1187	1174	0.2	0.03	trace	-	1589	1589	0.1	0.01	trace	-	[42]
31	*n*-dodecane	1200	1200	0.1	0.01	0.1	0.17	1200	1200	trace	-	0.2	0.09	
32	γ-terpineol	1204	1199	0.2	0.09	-	-	1709	-	trace	-	-	-	-
33	isomer of compound 29	1207	-	0.1	0.02	-	-	1771	-	trace	-	-	-	-
34	*n*-decanal	1215	1201	0.4	0.05	0.1	0.02	1491	1493	0.4	0.03	0.5	0.14	[43]
35	*trans*-piperitol	1218	1207	0.1	0.05	-	-	1736	1738	0.1	0.04	-	-	
36	pulegone	1228	1233	0.2	0.03	-	-	-	-	-	-	-	-	-
37	*exo*-fenchyl acetate	1230	1229	-	-	0.1	0.02	1457	1458	-	-	0.6	0.15	[44]
38	(2*E*)-decenal	1272	1260	0.7	0.09	0.2	0.02	1630	1630	0.6	0.08	0.4	0.04	[36]
39	1-tridecene	1292	1290	0.1	0.02	-	-	1351	1352	0.1	0.01	-	-	[45]
40	(2*E*,4*Z*)-decadienal	1306	1292	0.1	0.01	-	-	1753	1793	0.3	0.08	-	-	[46]
41	*p*-vinyl guaiacol	1324	1309	2.0	0.17	1.4	0.14	2186	2187	2.2	0.15	0.8	0.35	[47]
42	(2*E*,4*E*)-decadienal	1331	1315	0.2	0.04	0.4	0.02	1794	1795	0.4	0.03	0.7	0.03	[48]
43	α-cubebene	1347	1348	0.1	0.02	-	-	1521	1521	0.1	0.05	-	-	[49]
44	*α*-ylangene	1376	1373	0.4	0.01	1.4	0.14	1472	1472	0.3	0.02	1.4	0.19	[23]
45	β*-*bourbonene	1384	1387	-	-	1.2	0.28	1487	1491	-	-	0.8	0.05	[26]
46	(*E*)-β-damascenone	1386	1383	0.2	0.04	0.2	0.04	1802	1802	0.1	0.05	0.4	0.11	[50]
47	β-cubebene	1389	1387	-	-	trace	-	1469	1468	-	-	0.2	0.04	[51]
48	β-Elemene	1391	1389	0.5	0.04	-	-	1597	1596	s.p. 123	-	-	-	[52]
49	*n*-tetradecane	1400	1400	0.2	0.01	0.4	0.04	1400	1400	0.3	0.03	0.1	0.02	
50	α-gurjunene	1407	1409	0.3	0.02	-	-	1508	1507	trace	-	-	-	[53]
51	(*E*)-β-caryophyllene	1422	1417	13.6	1.42	7.0	0.77	1574	1575	14.5	1.1	7.8	1.56	[33]
52	β-copaene	1432	1430	0.1	0.00	0.2	0.02	1521	1522	0.1	0.02	0.0	0.01	[23]
53	sesquisabinene	1456	1457	0.1	0.02	-	-	1661	1648	s.p. 65	-	-	-	[54]
54	unidentified (MW = 204)	1458	1452	-	-	1.8	0.22	1271	-	-	-	1.9	0.57	-
55	α-humulene	1458	1452	1.1	0.07	-	-	1644	1644	1.1	0.11	-	-	[42]
56	allo-aromadendrene	1461	1458	0.1	0.01	0.1	0.02	1655	1655	0.2	0.02	0.1	0.03	[55]
57	*trans*-cadina-1(6),4-diene	1465	1475	-	-	0.1	0.02	1505	-	-	-	trace	-	-
58	9-*epi*-(*E*)-caryophyllene	1466	1464	0.2	0.01	-	-	1568	1572	0.1	0.01	-	-	[56]
59	4,5-di-*epi*-aristolochene	1473	1471	0.2	0.02	0.2	0.14	1657	1665	s.p. 57	-	0.1	0.03	[57]
60	β-chamigrene	1476	1476	0.2	0.04	-	-	-	-	-	-	-	-	-
61	*γ-*gurjunene	1478	1475	-	-	0.4	0.09	-	-	-	-	-	-	-
62	germacrene D	1485	1480	22.3	2.86	9.5	1.02	1685	1685	22.1	2.82	12.9	2.10	[42]
63	(*E*)-β-ionone	1488	1487	-	-	1.3	0.18	1883	1889	-	-	0.8	0.04	[58]
64	*cis*-β-guaiene	1491	1492	0.4	0.12	-	-	1669	1667	0.3	0.03	-	-	[59]
65	widdra-2,4(14)-diene	1491	1481	-	-	0.4	0.12	1554	-	-	-	0.6	0.10	-
66	*α*-zingiberene	1494	1493	-	-	1.2	0.12	1694	1696	-	-	2.4	0.57	[60]
67	γ-amorphene	1496	1495	0.1	0.02	-	-	-	-	-	-	-	-	-
68	bicyclogermacrene	1499	1500	2.6	0.34	1.0	0.19	1709	1707	2.3	0.32	1.2	0.25	[61]
69	α-muurolene	1503	1500					1705	1700					[62]
70	(*E*,*E*)-α-farnesene	1508	1505	0.4	0.05	0.4	0.03	1743	1743	0.3	0.06	0.2	0.03	[33]
71	β-bisabolene	1511	1505	0.3	0.03	-	-	1713	1710	s.p. 69	-	-	-	[49]
72	germacrene A	1511	1508	-	-	0.7	0.33	-	-	-	-	-	-	-
73	γ-cadinene	1518	1513	0.3	0.03	0.2	0.11	1738	1738	0.4	0.05	0.1	0.04	[42]
74	*n*-tridecanal	1519	1509	0.3	0.04	-	-	1806	1805	0.2	0.04	-	-	[43]
75	δ-cadinene	1522	1522	1.4	0.15	1.4	0.18	1738	1737	1.5	0.17	1.1	0.25	[26]
76	*trans*-cadina-1,4-diene	1538	1533	0.1	0.02	-	-	-	-	-	-	-	-	-
77	(*E*)-nerolidol	1567	1561	0.2	0.02	-	-	2036	2033	0.2	0.01	-	-	[63]
78	germacrene D-4-ol	1584	1574	1.4	0.17	-	-	2042	2044	0.1	0.01	-	-	[64]
79	spathulenol	1585	1577			3.6	0.66	2110	2106	1.3	0.26	3.2	0.19	[26]
80	caryophyllene oxide	1589	1582	3.0	0.44	7.2	1.29	1948	1944	3.1	0.32	6.7	0.75	[65]
81	*n*-hexadecane	1600	1600	0.3	0.03	0.5	0.04	1600	1600	0.3	0.02	0.2	0.05	
82	ledol	1612	1602	0.2	0.03	-	-	2000	2007	0.2	0.02	-	-	[66]
83	unidentified (MW = 222)	1613	-	-	-	0.8	0.02	2181	-	-	-	0.9	0.05	-
84	β-oplopenone	1616	1607	0.2	0.01	-	-	2039	2051	0.1	0.02	-	-	[66]
85	humulene epoxide II	1619	1608	-	-	1.3	0.04	1964	1972	-	-	0.8	0.12	[67]
86	*n*-tetradecanal	1621	1611	0.4	0.13	-	-	1911	1910	trace	-	-	-	[68]
87	allo-aromadendrene epoxide	1627	1639	0.4	0.03	0.4	0.02	2093	2095	0.2	0.02	0.2	0.05	[69]
88	unidentified (MW = 220)	1628	-	-	-	0.7	0.07	1940	-	-	-	0.8	0.07	-
89	junenol	1630	1618	0.2	0.03	-	-	-	-	-	-	-	-	-
90	1-*epi*-Cubenol	1636	1627	0.3	0.04	0.4	0.02	2052	2046	0.3	0.06	-	-	[70]
91	*cis*-cadin-4-en-7-ol	1644	1635	0.2	0.03	-	-	2090	-	0.2	0.04	-	-	-
92	*epi*-α-cadinol	1652	1638	0.5	0.24	0.7	0.06	2153	2154	0.8	0.13	0.6	0.09	[71]
93	*epi*-α-muurolol	1654	1640	0.4	0.30	0.6	0.05	2170	2171	0.7	0.11	0.5	0.20	[59]
94	α-muurolol (=torreyol)	1658	1645	-	-	0.7	0.37	2150	2150	-	-	0.5	0.20	[67]
95	7-*epi*-α-eudesmol	1657	1662	0.3	0.04	-	-	-	-	-	-	-	-	-
96	unidentified (MW = 220)	1660	-	-	-	0.7	0.05	2243	-	-	-	0.5	0.12	-
97	α-cadinol	1666	1652	1.6	0.22	2.1	0.13	2211	2211	2.0	0.27	3.0	0.29	[72]
98	*α*-amyl cinnamyl alcohol	1672	1682	-	-	0.8	0.15	2010	-	-	-	0.2	0.02	-
99	ar-turmerone	1673	1668	0.1	0.02	-	-	-	-	-	-	-	-	-
100	*n*-heptadecane	1700	1700	0.3	0.03	0.6	0.07	-	-	-	-	0.2	0.03	-
101	amorpha-4,9-dien-2-ol	1703	1700	0.7	0.07	1.2	0.24	2343	-	0.6	0.03	1.2	0.90	-
102	*n*-pentadecanal	1725	1724	0.7	0.06	1.0	0.05	2031	2024	0.7	0.19	0.4	0.09	[73]
103	1-octadecene	1795	1789	-	-	0.5	0.02	1831	1823	-	-	0.2	0.02	[74]
104	*n*-octadecane	1800	1800	-	-	0.5	0.06	1800	1800	-	-	0.2	0.01	-
105	cyclopentadecanolide	1831	1832	0.1	0.02	-	-	2255	2255	0.1	0.01	-	-	[75]
106	1-nonadecene	1895	1895	-	-	0.7	0.03	1934	1938	-	-	0.8	0.08	[76]
107	*n*-nonadecane	1900	1900	0.1	0.05	0.8	0.05	1900	1900	0.1	0.01	0.8	0.06	-
108	(5*E*,9*E*)-farnesyl acetone	1921	1913	0.1	0.06	-	-	2368	2364	0.2	0.03	-	-	[77]
109	1-eicosene	1996	1987	0.2	0.15	1.4	0.10	2057	2047	0.1	0.01	1.3	0.12	[78]
110	*n*-eicosane	2000	2000	0.1	0.10	0.6	0.05	2000	2000	trace	-	2.2	0.10	-
111	unidentified (MW = 270)	2095	-	-	-	2.4	0.24	2136	-	-	-	2.3	0.26	-
112	*n*-heneicosane	2100	2100	0.3	0.10	3.6	0.59	2100	2100	0.3	0.06	3.5	0.30	
113	1-docosene	2196	2189	0.1	0.03	4.0	0.46	2234	-	0.2	0.08	4.3	0.95	-
114	*n*-docosane	2200	2200	-	-	1.3	0.21	2200	2200	-	-	1.6	0.18	
115	1-tricosene	2297	2289	-	-	1.3	0.29	2235	-	-	-	1.6	0.21	-
116	*n*-tricosane	2300	2300	0.5	0.17	4.9	0.94	2300	2300	0.4	0.01	4.9	1.46	
117	1-teracosene	2397	-	-	-	1.4	0.32	2439	-	-	-	1.8	0.20	-
118	*n*-tetracosane	2400	2400	0.1	0.01	0.5	0.12	2400	2400	0.2	0.06	0.6	0.15	
119	1-pentacosene	2497	2486	-	-	0.4	0.09	2547	-	-	-	0.6	0.13	-
120	*n*-pentacosane	2500	2500	-	-	1.2	0.41	2500	2500	-	-	0.7	0.56	
121	1-hexacosene	2597	2596 *	0.3	0.04	-	-	2655	-	0.3	0.01	0.6	0.15	-
122	*n*-hexacosane	2600	2600	0.2	0.02	trace	-	2600	2600	0.2	0.02	0.6	0.20	
123	*n*-tetracosanal	2637	2650	0.1	0.01	-	-	2778	-	0.1	0.04	-	-	-
	monoterpenes			16.4		7.4				15.8		5.7		
	oxygenated monoterpenoids			2.3		0.7				1.4		1.0		
	sesquiterpenes			44.8		27.5				43.4		31.3		
	oxygenated sesquiterpenoids			9.9		20.4				10.0		18.9		
	others			13.0		34.2				12.2		34.3		
	total			86.3		90.2				82.8		91.2		

LRI—Linear Retention Index; Calc.—Calculated; Ref.—Reference; Lit.—Literature; %—Percent amount by weight; σ— Standard deviation; MW—Molecular Weight; * Reference [79].

**Table 2:** Enantioselective analyses of *G. reinaldii* and *G. pulchella* EOs on 2,3-diacetyl-6-*tert*-butyldimethylsilyl-β-cyclodextrin and 2,3-diethyl-6-*tert*-butyldimethylsilyl-β-cyclodextrin stationary phases.

Enantiomers	LRI	*G. reinaldii*		*G. pulchella*	
		Composition	ee (%)	Composition	ee (%)
(1*S*,5*S*)-(−)-α-pinene	926 ^a^	100.0	100.0	12.0 *	76.0
(1*R*,5*R*)-(+)-α-pinene	928 ^a^	-		88.0 *	
(1*R*,5*R*)-(+)-β-pinene	949 ^b^	-	100.0	96.9	93.8
(1*S*,5*S*)-(−)-β-pinene	959 ^b^	100.0		3.1	
(1*R*,5*R*)-(+)-sabinene	1008 ^a^	-	100.0	66.7	33.4
(1*S*,5*S*)-(−)-sabinene	1014 ^a^	100.0		33.3	
(*R*)-(−)-α-phellandrene	1019 ^b^	100.0	100.0	100.0	100.0
(*S*)-(+)-α-phellandrene	1024 ^b^	-		-	
(*R*)-(−)-β-phellandrene	1051 ^b^	100.0	100.0	-	-
(*S*)-(+)-β-phellandrene	1058 ^b^	-		-	
(*R*)-(−)-*cis*-linalool oxide (furanoid)	1209 ^a^	40.7	18.6	32.4	35.2
(*S*)-(+)-*cis*-linalool oxide (furanoid)	1197 ^a^	59.3		67.6	
(*R*)-(−)-linalool	1300 ^a^	51.5	3.0	62.7	25.4
(*S*)-(+)-linalool	1301 ^a^	48.5		37.3	
(*R*)-(−)-terpinen-4-ol	1338 ^a^	65.1	30.2	-	-
(*S*)-(+)-terpinen-4-ol	1379 ^a^	34.9		-	
(*R*)-(+)-germacrene D	1466 ^b^	98.8	97.6	98.4	96.8
(*S*)-(−)-germacrene D	1471 ^b^	1.2		1.6	

^a^ 2,3-diacetyl-6-tert-butyldimethylsilyl-β-cyclodextrin; ^b^ 2,3-diethyl-6-tert-butyldimethylsilyl-β-cyclodextrin; * partially resolved.

## Data Availability

The datasets presented in this article are not readily available because they are part of an ongoing study. Requests to access the datasets should be directed to the corresponding author.
